# The supplementation of lubabegron or ractopamine hydrochloride in the summer improved growth performance but did not impact rumination, mobility scores, and behaviors in feedlot cattle in the United States^†^

**DOI:** 10.1093/jas/skag114

**Published:** 2026-04-10

**Authors:** Huey Yi Loh, Paxton A Sullivan, Jeniffer Backes-Kincher, Michelle Calvo-Lorenzo, Terry E Engle, Lily Edwards-Callaway

**Affiliations:** Department of Animal Sciences, Colorado State University, Fort Collins, CO 80523, United States; Department of Animal Sciences, Colorado State University, Fort Collins, CO 80523, United States; Department of Animal Sciences, Colorado State University, Fort Collins, CO 80523, United States; Elanco Animal Health, Indianapolis, IN 46221, United States; Department of Animal Sciences, Colorado State University, Fort Collins, CO 80523, United States; Department of Animal Sciences, Colorado State University, Fort Collins, CO 80523, United States

**Keywords:** β-agonist, β-ligands, behavior, heat stress, welfare

## Abstract

This study evaluated the effects of β-ligand supplementation on growth performance, carcass characteristics, rumen parameters, mobility, and behavior in feedlot steers finished in the summer months. Three hundred sixty Angus crossbred steers were ranked by body weight (BW) and randomly assigned to one of three treatments: 1) Control (no β-ligand), 2) Ractopamine Hydrochloride (RAC; 300 mg·animal^−1^·d^−1^), or 3) Lubabegron (LUB; 36 mg·animal^−1^·d^−1^). RAC and LUB were fed for the final 35 d of the finishing period, followed by a 4 d voluntary withdrawal period prior to slaughter. Steers were housed in feedlot pens (10 head/pen; 12 pens/treatment). A steam flaked corn-based diet with the appropriate treatment was delivered once daily to provide cattle *ad libitum* access to feed over a 24 h period. Body weights and mobility scores were obtained at the beginning of the experiment and every 14 d throughout the supplementation period. Weekly in-pen behavior was observed via 24 h video footage, using instantaneous sampling with a 10-minute scan interval. Rumen boluses were administered to 2 steers per pen before the initiation of the experiment to continuously monitor reticulorumen temperature, pH, and rumination events. Ambient environmental conditions were recorded throughout the study. Post-slaughter carcass characteristics were evaluated. The RAC and LUB supplemented steers had greater (*P *< 0.05) final BW, average daily gains, and feed efficiencies compared to controls. Steers supplemented with RAC and LUB had greater (*P *< 0.05) hot carcass weights and longissimus muscle areas than control steers. No treatment differences (*P *> 0.05) were detected for dressing percentage, quality grade, subcutaneous fat depth, USDA yield grade, and meat color. There was no treatment effect (*P *> 0.10) for reticulorumen pH, temperature, or rumination time. Similar (*P *> 0.05) mobility score distributions were observed across treatments. The majority of cattle exhibited normal mobility over the course of the study. Three-way interactions (*P *< 0.01) between treatment, week, and time of day were observed for drinking, standing, and lying behaviors. A two-way interaction (*P *< 0.05) between treatment and week was detected for locomotion behavior. In summary, RAC and LUB supplementation to feedlot steers during the summer months improved growth performance and carcass characteristics and had no impact on mobility scores, rumen parameters, or behaviors when compared to control steers.

## Teaser

This study evaluated the effects of β-ligand supplementation during the summer months and observed improved feedlot performance and carcass characteristics in cattle that received β-ligand supplementation. Mobility scores, behaviors, and rumination parameters were similar between control and β-ligand supplemented steers.

## Introduction

Beta-adrenergic ligands (broadly termed as β-ligands, also commonly known as β-agonists; β-AA) have been utilized in the US cattle feeding industry for over two decades as approved growth promoters to improve feed efficiency and increase lean tissue deposition (FDA 2000; FDA 2006). The supplementation of β-ligands to feedlot cattle is shown to increase average daily gain (ADG), gain-to-feed ratio (G:F), hot carcass weight (HCW), dressing percentage, and longissimus muscle area (Abney et al. 2007; Gruber et al. 2007; Strydom et al. 2009; Bryant et al. 2010). In 2018, another β-ligand, lubabegron (LUB, marketed as Experior by Elanco Animal Health, Indianapolis, IN, USA), was approved by the FDA with a claim of “reduction of ammonia gas emissions per pound of live weight and hot carcass weight in growing beef steers and heifers fed in confinement for slaughter during the last 14–91 days” (FDA-FOI 2018; Putnam 2018). Although LUB has no label claim for an improvement in feed efficiency and lean muscle gain, previous studies have shown improved feedlot performance and heavier carcass weights in cattle fed LUB compared to cattle that did not receive LUB (FDA-FOI 2018; Kube et al. 2021; Teeter et al. 2021; Edmonds et al. 2024).

While the use of β-ligands contributes to improved production efficiency, sustainability, and positive return on investment, concerns have been raised regarding their potential impact on animal welfare, especially during hot weather (Grandin 2013; Smith 2023). Increased incidences of lameness and heat stress symptoms have been observed at slaughter in fed cattle, some of which have been fed high doses of β-AA during elevated ambient temperatures during the summer months (Grandin 2013; Grandin and Whiting 2018). In addition, β-AA supplementation to feedlot sheep was associated with increased glucose metabolism, suggesting that β-AA may elevate metabolic heat production, potentially worsening heat stress symptoms in animals already experiencing heat stress (Barnes et al. 2019; Sieck et al. 2021). The heat stress resulting from the combined effects of increased environmental temperatures and metabolic heat production can compromise cattle welfare and performance, and in severe cases, lead to mortality (Rhoads et al. 2013; Chappell 2022; Polansek 2023). Management strategies such as adding shade, using sprinklers, and changing feeding times have been evaluated to alleviate heat stress in cattle finished during the summer (Davis et al. 2003; Mader and Davis 2004; Rhoads et al. 2013). These management strategies were able to reduce some of the negative impacts from heat stress, but the decline in growth performance and cattle death were still being reported in cattle finished during the summer (Rhoads et al. 2013; Chappell 2022; Polansek 2023). Consequently, heat stress remains a major challenge for the beef industry, contributing to recurring cattle deaths during summer months and economic losses estimated at $369 million annually in the US (St-Pierre et al. 2003; Polansek 2023; Myers 2025).

Despite some of the concerns that have been raised on the utilization of β-ligands in feedlot cattle, limited research has examined their influence on behavioral and welfare outcomes in feedlot cattle, particularly during the summer months. There have been studies evaluating the impacts of β-AA on cattle behavior during handling events (Baszczak et al. 2006; Hagenmaier et al. 2017a, 2017b) and in home pens at the feedyard (Boyd et al. 2015; Hagenmaier et al. 2016; Trotta et al. 2019). However, to the authors’ knowledge, the in-pen behavior of cattle fed LUB has not yet been documented in the existing literature. Furthermore, limited to no published literature has investigated the effect of supplementing Ractopamine Hydrochloride (RAC, marketed as Optaflexx by Elanco Animal Health, Indianapolis, IN, USA) or LUB on performance, carcass traits, and welfare parameters of feedlot cattle during the summer months. Therefore, the objective of this study was to evaluate the impact of β-ligand supplementation during the summer months on feedlot performance, rumination parameters, carcass traits, mobility scores, and behavioral parameters in feedlot steers. We hypothesized that cattle receiving RAC or LUB would have improved feedlot performance, rumen parameters, and carcass traits compared to cattle that did not receive β-ligands, while mobility scores and behavior patterns would remain consistent across treatments.

## Materials and methods

### Ethical statement

Before experiment initiation, all animal care, handling, and use procedures described herein were approved by the Colorado State University Institutional Animal Care and Use Committee (CSU IACUC approval # 5253).

### Animals, diets, and treatment

Four hundred yearling Angus crossbred steers were sourced from a commercial feedlot and transported (approximately 960 km) to the Colorado State University Agriculture Research and Development and Education Center (ARDEC) feedlot in March 2024. Steers selected were uniform in weight and fatness (visual appraisal). Upon arrival, steers were individually ear-tagged for ID, weighed, vaccinated with Bovishield Gold (Zoetis, Parsippany, NJ, USA) and 7-Way Ultrachoice (Zoetis, Parsippany, NJ, USA), given Noromectin (NorBrook Labs, Lenexa, KS, USA), orally drenched with Synathic (Boehringer Ingelheim, St. Joseph, MO, USA) for parasite control, and implanted with Revalor–XS (200 mg trenbolone acetate and 40 mg estradiol, Merck Animal Health, Rahway, NJ). After processing, steers were provided *ad libitum* access to long-stem grass hay and water and were housed overnight (10 steers per pen). After initial weighing to obtain an arrival body weight (BW; mean arrival BW = 383 ± 21 kg), steers (*n* = 40) that were greater than ± 2 SD from the mean arrival BW were eliminated from further consideration in the experiment. The 360 black-hided Angus steers selected for study enrollment were ranked by BW and stratified into 3 weight blocks of 120 steers each (*n* = 3 blocks; block 1 = heaviest, block 3 = lightest). Within each weight block, steers were sequentially grouped by BW into 4 replicates of 30 steers (4 replicates/weight block, 12 replicates total across 3 weight blocks), such that the heaviest 30 steers within a weight block formed replicate 1, the next heaviest 30 formed replicate 2, and so forth. Within each replicate, steers were randomly assigned to 1 of 3 pens (*n* = 10 steers per pen). Pens were then randomly allocated to 1 of 3 treatments. This process was repeated until all steers within each weight block were assigned to a replicate, pen, and treatment (40 steers per treatment within each weight block, total of 120 steers for 3 treatments within each weight block). Treatments consisted of: 1) Control: no β-ligand supplementation, 2) RAC: Ractopamine Hydrochloride (Optaflexx 45, Elanco Animal Health, IN, USA) supplemented at 300 mg·animal^−1^·d^−1^and applied during the final 35 d on feed and 3) LUB: Lubabegron (Experior 50, Elanco Animal Health, IN, USA) supplemented at 36 mg·animal^−1^·d^−1^ and applied during the final 35 d on feed. A 4 or 5 d voluntary withdrawal period was applied for all treatments before slaughter (blocks 1 and 3 had a 5 d withdrawal and block 2 had a 4 d withdrawal). Steers were housed in soil-surfaced treatment pens (7 m × 40 m; 3% slope; *n *= 10 steers/pen; 24.4 m^2^/steer) equipped with a concrete feed bunk (3 m × 7 m concrete bunk pad) and heated automatic waterers (Cancrete C250; one time volume: 106 L; feeder cattle head capacity: 200–250 head) that were shared between two pens. Pens were monitored daily for illness, lameness, to ensure that cattle were in the correct assigned pen, and that all cattle had *ad libitum* access to feed and water.

Steers were transitioned to the finishing diet using a series of step-up diets (a total of 3 step-up diets). Diet changes during the step-up program were simultaneous (every 5–7 d) for all treatments, and all cattle were transitioned to the finishing diet by d 22 following cattle allotment to treatment. A steam flaked corn-based high-energy finishing diet (Table 1) was formulated to meet or exceed requirements for growing and finishing beef cattle (NASEM 2016). Treatments were supplemented using wheat midd-dried distillers’ grains-calcium carbonate-based carrier pellet manufactured by Scott Pro (Scott City, KS) and mixed with the basal diet. Diets were manufactured daily and delivered once daily in the morning (at approximately 0700 h) in amounts to allow *ad libitum* feed access over a 24 h period. Experimental rations were sampled weekly, stored at −20°C, and submitted for nutrient analysis as a monthly composite. Samples were assayed for proximate analysis using standard Association of Official Agricultural Chemist International Methods (AOAC 2016; SDK Laboratories, Inc., Hutchinson, KS). The analyzed nutrient profile for the basal control diet is reported in Table 1.

**Table 1:** Ingredient and nutrient composition of the basal control diet.

Item	
**Ingredient, % dry matter basis**	
** Steam flaked corn**	70.8
** Corn silage**	14.1
** Dried distillers grains**	7.9
** Liquid supplement^1^**	3.2
** Carrier pellet^2^**	4.0
**Chemical analysis**	Mean (± standard deviation)
** Dry matter, %**	65.70 (0.38)
** Crude protein, %**	13.93 (0.74)
** Acid detergent fiber, %**	8.38 (0.44)
** Neutral detergent fiber, %**	15.84 (0.53)
** NE_m_, Mcal/kg^3^**	1.99 (0.01)
** NE_g_, Mcal/kg^4^**	1.34 (0.02)
** Total starch, %**	56.12 (2.24)
** Crude fat, %**	3.72 (0.14)
** Calcium, %**	0.59 (0.05)
** Phosphorus, %**	0.38 (0.01)
** Potassium, %**	0.70 (0.07)
** Magnesium, %**	0.18 (0.007)
** Sodium, %**	0.12 (0.02)
** Chloride, %**	0.40 (0.01)
** Sulfur, %**	0.23 (0.02)
** Cobalt, mg/kg**	0.45 (0.27)
** Copper, mg/kg**	18.83 (2.2)
** Iron, mg/kg**	137.89 (17.84)
** Manganese, mg/kg**	62.07 (6.21)
** Molybdenum, mg/kg**	0.47 (0.13)
** Zinc, mg/kg**	110.2 (12.1)

1 Liquid supplement (molasses based suspension) at 3.2% inclusion in the diet provided: 3.5% NPN (Urea), 0.46% Ca (CaCO_3_), 0.22% Salt (NaCl), 0.03% K (KCl), 0.20 mg of Co/kg DM, 16 mg of Cu/kg DM, 32 mg of Mn/kg DM, 0.21 mg Se/kg DM, 53 mg of Zn/kg DM, 2,346.6 IU/kg Vitamin A, 18.1 IU/kg Vitamin E, 310.5 mg·animal^−1^·d^−1^ of monensin (Rumensin 90, Elanco Animal Health, Greenfield, IN), and 89.0 mg·animal^−1^·d^−1^ of tylosin (Tylan 100, Elanco Animal Health, Greenfield, IN).

2 Carrier pellet supplement was a wheat midd-dried distillers grains-calcium carbonate-based carrier that was used to supply control or β-ligand treatments for the final 35 d of the finishing period: Control (no β-ligands added), RAC (Optaflexx 45, Elanco Animal Health, IN, USA) supplemented at 300 mg·animal^−1^·d^−1^, and LUB (Experior 50, Elanco Animal Health, IN, USA) supplemented at 36 mg·animal^−1^·d^−1^.

3 Net energy for maintenance; {[1.37 × (TDN × 0.0361)] − [0.0138 × (TDN × 0.0361) × (TDN × 0.0361)] + [0.0105 × (TDN × 0.0361) × (TDN × 0.0361) − 1.12]} ÷ 2.205.

4 Net energy for gain; {[1.42 × (TDN × 0.0361)] − [0.174 × (TDN × 0.0361) × (TDN × 0.0361)] + [0.0122 × (TDN × 0.0361) × (TDN × 0.0361) × (TDN × 0.0361) − 1.65]} ÷ 2.205.

Steers consumed the basal control diet for approximately 4–5 months until the average weight of steers (*n* = 120) within each weight block (*n* = 3) reached approximately 665 ± 19 kg. After the steers reached their targeted BW, steers within each respective weight block began receiving the assigned dietary treatment (Control, RAC, or LUB). Treatments were initiated on July 10th, August 4th, and August 16th of 2024 for weight blocks 1, 2, and 3, respectively. Steers were individually weighed on d −1 (baseline), d 0 (the start of treatment supplementation), d 5 (Week 1), d 19 (Week 3), d 33 (Week 5), and on 2 consecutive d (final BW) at the termination of the trial prior to slaughter. On d 35 of the experiment, within each block, a subsample of steers (*n* = 3 per pen; *n* = 36 per block) from each pen underwent a simulated transport trial (data reported elsewhere). Information from these steers has been included in the current statistical analyses. Four steers were removed from the study because of complications unrelated to experimental treatments. In summary, one animal was euthanized due to blindness, two were euthanized for compromised mobility associated with injuries, and one animal died from bloat.

### Environmental condition monitoring

Environmental conditions were continuously monitored throughout the study using a Kestrel 5400AG Cattle Heat Stress Tracker (Nielsen-Kellerman Co., Boothwyn, PA, USA), predominantly beginning when cattle received their dietary treatments and through the end of the study (10 July 2024 to 19 September 2024). The Kestrel weather system was secured in an open area near the treatment pens to reflect ambient conditions experienced by the cattle. Ambient temperature (AT), black globe temperature (BGT), RH, WS, and temperature-humidity index (THI) were automatically recorded at 10-minute intervals. Weather data was summarized as daily and hourly [morning (0700 h to 0900 h), afternoon (1200 to 1400 h), and evening (1700 to 1900 h)] averages for days when cattle were receiving treatment diets. The heat load index (HLI) of every 10-minute interval was calculated using the equations proposed by Gaughan et al. (2008) using environmental conditions recorded at their respective intervals. When BGT exceeded 25 °C, HLI was calculated as: HLI = 8.62 + (0.38 × RH) + (1.55 × BGT) − (0.5 × WS) + e^(2.4 − WS)^. When BGT was ≤ 25 °C, the following equation was used: HLI = 10.66 + (0.28 × RH) + (1.3 × BGT) − WS. Ground temperature within each pen was also measured one time each week between 1200 h and 1530 h. Ground temperature measurement began on week 5 of block 1; therefore, only one week of data is reported for block 1, whereas complete data were obtained for blocks 2 and 3. A laser infrared thermometer (Shenzhen Mestek Electronics Co., LTD, Shenzhen, China) was used to take 3 ground temperature measurements between 1200 h and 1530 h inside each pen one day per week. Location of the measurements included approximately 0.61 m from the concrete bunk pad, in the middle of the pen, and 0.61 m from the back of the pen. These values were averaged to calculate a mean pen ground temperature for each time point.

### Rumen measurements

Before treatment supplementation, seventy-two steers (*n* = 2 steers per pen) were randomly selected based on similar BW across replicates to receive a reticulorumen bolus (SmaXtec pH bolus and Base Station SX.2; SmaXtec GmbH, Graz, Austria). Before bolus application, boluses were assigned to their respective cattle, connected to the base station to facilitate data collection, and calibrated using the manufacturer-provided calibration solution (pH = 7) following the bolus manual guidelines. Cattle were restrained in a chute to minimize movement during bolus administration. The assigned bolus was placed into a manufacturer-provided bolusing device, inserted into the steer’s mouth, and guided down the esophagus. The animal was then allowed to swallow the bolus naturally before the device was removed. Throughout the study period, boluses continuously measured reticulorumen temperature, reticulorumen pH, adjusted reticulorumen temperature, and rumination (Gasteiner et al. 2009). Data were recorded at 10-minute intervals throughout all study days; data from the boluses were transmitted to the base station and made available on the online platform for download.

### Mobility and behavior

Cattle mobility was scored using the North American Meat Institute’s (NAMI) mobility scoring system (2015) for finished cattle. The scoring system categorizes mobility as follows: 1 = normal, walks easily, no apparent lameness, no change in gait; 2 = minor stiffness, shortness of stride, slight limp, keeps up with normal cattle; 3 = obvious stiffness, difficulty taking steps, obvious limp, obvious discomfort, lags behind normal cattle; 4 = extremely reluctant to move even when encouraged, statue-like. To score mobility, video recordings were obtained of study cattle on the same days that cattle were weighed (Baseline = d −1, week 1 = d 5, week 3 = d 19, and week 5 = d 33). Cameras (GoPro Hero10; GoPro Inc., San Mateo, CA, USA) were mounted in the drive alley to capture cattle movement from their home pen to the working facility to be weighed. One trained observer, with extensive experience scoring finished cattle mobility, assessed the movement of all study cattle from the camera footage and recorded mobility scores for each individual animal.

To evaluate daily cattle behaviors, video footage of cattle in each pen was obtained using pen-mounted video cameras (Reolink RLC-81PA, Reolink Innovation Limited, Mong Kok, Hong Kong), which continuously recorded cattle activity the day before BW were obtained each week. The video was recorded on d −1 (baseline, week 0), d 4 (week 1), d 11 (week 2), d 18 (week 3), d 25 (week 4), and d 32 (week 5). Behavior was recorded for 3 time periods of the day: 1) Morning (0700 h to 0900 h), 2) Afternoon (1200 h to 1400 h), and 3) Evening (1700 h to 1900 h). Video footage was stored on a network video recorder (Reolink Network Video Recorder RLNB-410, Reolink Innovation Limited, Mong Kok, Hong Kong). Cattle behavior video footage was watched by 2 trained observers using instantaneous sampling methods of a 10-minute scan interval (Bateson and Martin 2021). Reliability tests were conducted to achieve at least 80% inter-rater reliability between observers before the videos were watched. At each interval scan, the number of steers performing a specific behavior was tallied so that the data represent the percentage of steers performing a behavior during a specified time period. An ethogram with five behaviors of interest to be observed (Table 2) was adapted from Park et al. (2020) and Meneses et al. (2021). Due to the view of the pen obtained with video recorders, feeding and drinking behavior were defined as animals performing the behaviors in addition to those within one body length from the feed bunk and the waterer.

**Table 2:** Pen behaviors of interest and their definition.

Behavior^1^	Definition
**Feeding**	Steer facing the feed bunk and within one body length of the feed bunk. Within this behavior category the steer could have been eating or standing within the proximity described and not eating.
**Drinking**	Steer facing the water trough and within one body length of the water trough. Within this behavior category the steer could have been drinking or standing within the proximity described and not drinking.
**Standing**	Steer in an upright position supported by all four legs
**Lying**	Steer in a recumbent position not supported by its legs
**Locomotion**	Steer taking at least two consecutive steps to move its body forward or backward

1 Behavior definition was adapted from Bateson and Martin (2021).

### Carcass measurements

Study cattle within a weight block were transported to a commercial beef processing facility in Colorado, located 16 km away from the research facility, on 3 separate slaughter days. Trained researchers performed tag transfer to obtain the kill order of carcasses and associated carcass IDs. The commercial plant personnel collected all carcass characteristics following their standard operating procedures and shared the data with the university. The carcasses were chilled for 24–36 h. A 4% pencil shrink was applied to the final live body weight to determine the final shrunk body weight. Dressing percentage was calculated by dividing hot carcass weight by final shrunk body weight and multiplying by 100. Marbling score was enumerated using instrument grading systems for beef carcasses with the following scale: 300 = Slight, 400 = Small, 500 = Modest, 600 = Moderate, and 700 = Slightly Abundant (USDA 2006). A proprietary camera-based system was used to assess meat color during processing at the plant, categorizing samples based on objective color scores. The scoring thresholds were as follows: a score of < 55 indicated a ‘Dark Cutter,’ scores between 55 and 59 represented ‘Off-Colored’ meat, and scores ≥ 60 were classified as ‘Normal’ meat color.

### Statistics

All statistical analyses were conducted using R Statistical Software (version 4.4.2, 2024-10-31; R Core Team 2023). Outlier detection was performed on all data, and values exceeding ± 3 standard deviations (SD) from the mean were excluded from the analysis. The best fit model was selected based on Akaike’s Information Criterion (AIC). Significance for all analyses was assessed at *P* ≤ 0.05.

#### Environmental conditions, feedlot performance, carcass characteristics, and mobility

All environmental parameters recorded during the treatment period (10 July 2024 to 19 September 2024) were grouped by time of day and day, and mean ± SD, minimum, and maximum values were computed using the summarize function in R (dplyr package). Because all treatments (Control, RAC, and LUB) within each block were housed concurrently and exposed to the same environmental conditions (4 pens per treatment; 12 pens per weight block), environmental measurements were not associated with individual treatments or pens. Therefore, these data were summarized descriptively to provide context for the study rather than analyzed statistically for treatment effects.

Feedlot performance, rumen measurement, and carcass data were analyzed on a pen mean basis, with pen serving as the experimental unit. For all measured variables, linear mixed-effects models were fitted with treatment as a fixed effect and weight block as a random effect. Pen initial BW was used as a covariate in the analysis of all performance and carcass response variables except for initial BW. A Type II Analysis of Variance (ANOVA) table was generated using the Kenward-Roger method to calculate denominator degrees of freedom. Statistical differences between treatment groups were computed using the emmeans package (Lenth et al. 2024). As all carcasses were classified as either Choice or Prime, the QG of carcass data was analyzed as binomial outcomes. As no cattle received a mobility score of 3 or greater, mobility scores were treated as binomial factors, either 0 (normal; mobility score of 1) or 1 (abnormal; mobility score of 2). A generalized linear mixed model using Template Model Builder (R package: glmmTMB) was fitted for the number of steers within pens having a normal mobility score as the outcome of the interaction between treatment and weeks of receiving treatment. Pen was the experimental unit for mobility scores and was included as a random effect in the model.

##### Behavior data

For behavior data, a glmmTMB was fitted with the number of steers exhibiting certain behaviors (feeding, drinking, lying, standing, and locomotion; Table 2) as the outcome and the interaction between treatment, week (treatment length; weeks 0–5), and time period (morning, afternoon, and evening); these behavior data served as the predictor, accounting for the total steers captured within the pen as the offset. Weight block and pen were included as random effects. A likelihood ratio test was used to test for the 3-way interactions from this mixed logistic regression model. If the 3-way interaction was significant (*P* ≤ 0.05), then the marginal means (least squares means) differences between treatment groups were computed using emmeans. If the 3-way interaction was not significant (*P* > 0.05), then 2-way interactions were the focus and the statistical differences between treatment groups were computed using emmeans. Graphs with unique symbols indicating differences between treatments were generated using the emmeans and ggplot2 packages. Observations with less than 50% of the total steers captured within a pen were removed from the analysis. In some cases, steers’ behavior could not be determined when steers in the pen were bunched together and only a portion of the steers were visible; if fewer than half of the steers’ behavior could be identified, the data point was excluded. A total of 760 out of 8,425 observations were removed for behavior data.

## Results

### Environmental conditions

Descriptive weather data are summarized in Table 3 without statistical comparison, as all steers were exposed to the same environmental conditions. Daily mean weather parameters were 21.53 °C (AT), 25.78 °C (BGT), RH of 54.24, WS of 1.86 m/s, THI of 66.23, and HLI of 48.87. Mean ambient temperature, BGT, and WS were numerically greater in the afternoon, followed by the evening and morning. The THI was the greatest in the evening numerically, followed by the afternoon and the morning. Morning RH was the highest numerically, while afternoon RH was the lowest. Heat load index was the highest in the afternoon numerically, followed by the morning, and then the evening. The mean pen ground temperature during the treatment period was 39.67 ± 13.24 °C, with a minimum temperature of 16.28 °C and a maximum temperature of 62.11 °C. Across 5,032 (block 1), 4,527 (block 2), and 4,531 (block 3) 10-min observations, 20.3%, 13.48%, and 9.69% of THI observations and 1.93%, 1.52%, and 0.68% of HLI observations exceeded thermoneutral thresholds in blocks 1, 2, and 3, respectively.

**Table 3:** Ambient temperature, black globe temperature, relative humidity, wind speed, temperature-humidity index (THI), and heat load index (HLI) at morning (0700 to 0900 h), afternoon (1200 to 1400 h), evening (1700 to 1900 h), and daily averages for days when cattle were on treatment diets.

Item	Mean ± standard deviation		
	Block 1	Block 2	Block 3
**Morning (0700 to 0900 h) **			
** Temperature, °C**			
** Ambient**	20.20 ± 3.75	18.39 ± 4.34	16.92 ± 4.61
** Black globe**	27.82 ± 6.83	24.69 ± 6.70	23.29 ± 6.26
** Relative humidity, %**	65.19 ± 22.12	70.58 ± 20.74	62.83 ± 16.90
** Wind speed, m/s**	1.55 ± 1.16	1.35 ± 1.03	1.34 ± 0.91
** THI^1^**	65.77 ± 4.70	63.44 ± 5.87	61.18 ± 6.38
** THI > 75, %**	0.88	0.74	0.25
** HLI^2^**	52.75 ± 7.58	52.12 ± 7.71	47.15 ± 6.44
** HLI > 70, %**	1.76	1.49	0
**Afternoon (1200 to 1400 h) **			
** Temperature, °C**			
** Ambient**	29.28 ± 4.95	27.63 ± 4.83	28.09 ± 3.27
** Black globe**	40.18 ± 8.07	38.23 ± 7.48	3,894 ± 4.35
** Relative humidity, %**	38.60 ± 21.33	41.43 ± 20.27	31.61 ± 8.61
** Wind speed, m/s**	2.71 ± 1.53	2.61 ± 1.32	2.65 ± 1.25
** THI^1^**	74.73 ± 4.84	73.26 ± 4.99	73.18 ± 3.17
** THI > 75, %**	60.66	38.22	26.92
** HLI^2^**	58.48 ± 7.80	55.91 ± 8.66	50.52 ± 7.85
** HLI > 70, %**	4.62	3.37	0.72
**Evening (1700 to 1900 h)**			
** Temperature, °C**			
** Ambient**	27.72 ± 5.48	26.60 ± 4.88	26.89 ± 3.70
** Black globe**	32.80 ± 8.09	31.43 ± 7.52	31.21 ± 6.44
** Relative humidity, %**	45.45 ± 29.28	45.10 ± 25.69	34.01 ± 14.62
** Wind speed, m/s**	2.61 ± 1.95	2.09 ± 1.51	1.93 ± 1.43
** THI^1^**	73.33 ± 4.98	72.21 ± 4.76	71.95 ± 3.62
** THI > 75, %**	46.22	29.3	19.61
** HLI^2^**	52.59 ± 11.48	51.07 ± 8.90	46.16 ± 7.51
** HLI > 70, %**	4.67	1.45	0.73
**Daily**			
** Temperature, °C**			
** Ambient**	22.44 ± 6.76	21.16 ± 6.57	20.63 ± 7.01
** Black globe**	26.83 ± 11.34	25.17 ± 10.79	24.66 ± 11.26
** Relative humidity, %**	57.00 ± 26.05	60.37 ± 24.02	51.28 ± 21.71
** Wind speed, m/s**	1.98 ± 1.52	1.75 ± 1.40	1.76 ± 1.39
** THI^1^**	67.49 ± 7.30	66.11 ± 7.59	64.98 ± 8.18
** THI > 75, %**	20.29	13.47	9.69
** HLI^2^**	51.56 ± 8.77	50.89 ± 8.21	46.16 ± 7.90
** HLI > 70, %**	1.92	1.52	0.68
**Pen ground temperature^3^, °C**	31.53 ± 3.64	36.16 ± 14.56	45.22 ± 10.98

1 Temperature-humidity index (THI) = 0.8 × ambient temperature + [(% relative humidity ÷ 100) × (ambient temperature − 14.4)] + 46.4, adapted from Arias and Mader (2023). THI categories: THI < 75 = Normal; THI 75–78 = Alert; THI 79–83 = Danger; THI > 84 = Emergency. THI > 75 (%): Proportion of THI observations (10-minute interval) exceeding 75 (normal) within each time point and within each day.

2 HLI = Heat load index; HLI_BG<25°C_ = 10.66 + (0.28 × relative humidity) + (1.3 × black globe temperature) – windspeed and HLI_BG>25°C_ = 8.62 + (0.38 × relative humidity) + (1.55 × black globe temperature) − (0.5 × windspeed) + [e^(2.4−WS)^], where e = the base of natural logarithm, adapted from Gaughan et al. (2008). HLI categories: HLI ≤ 70 = Thermoneutral, no heat stress; 70.1 ≤ HLI ≤ 77 = Warm, mild heat stress; 77.1 ≤ HLI ≤ 86 = Hot, moderate heat stress; HLI ≥ 86 = Very hot, severe heat stress. THI > 75 (%): Proportion of THI observations (10-minute interval) exceeding 70 (thermoneutral) within each time point and within each day.

3 Pen ground temperature was measured using a laser infrared thermometer (Shenzhen Mestek Electronics Co.,LTD, Shenzhen, China) on each heart rate data collection day between 1200 h and 1530 h in pens housing cattle wearing heart rate monitors. Location of the measurements included 0.61 m away from the concrete bunk pad, in the middle of the pen, and 0.61 m away from the back of the pen. Data was collected one day per week.

### Feedlot performance and rumen measurement

The influence of β-ligand supplementation on growth performance in finishing beef cattle is reported in Table 4. There were no differences (*P *> 0.05) across treatments in initial BW and DMI. Steers that received RAC and LUB had greater (*P *< 0.05) final BW, ADG, and feed efficiency than the control steers, but there were no differences (*P *> 0.05) between these parameters in the RAC and LUB steers. Supplementing β-ligand did not influence reticulorumen pH, temperature, or rumination (*P *> 0.10; Table 5) in β-ligand supplemented steers when compared to the control steers.

**Table 4:** Impact of β-ligand supplementation for the last 35 d on feed on growth performance, carcass characteristics, and muscle color in finishing beef cattle.^1^

Item	Treatment^2^			SEM^4^	*P*-Value
	Control	RAC	LUB		
**Pens**	12	12	12	—	—
**Animals**	120	120	120	—	—
**Initial body weight^3^, kg**	668.1	665.3	664.3	5.50	0.86
**Final body weight, kg **	742.1^b^	747.9^a^	753.0^a^	2.98	<0.01
**Average daily gain, animal^−1^·d^−1^**	2.18^b^	2.35^a^	2.49^a^	0.09	<0.01
**Dry matter intake, kg/animal^−1^·d^−1^**	11.83	11.64	11.85	0.24	0.77
**Feed efficiency, gain:feed**	0.184^b^	0.202^a^	0.210^a^	0.006	0.01

1 Initial body weight was used as a covariate for statistical analysis of all response variables with the exception of initial body weight. Pen was the experimental unit for statistical analysis.

2 Treatment: Control: No added β-ligand; RAC: Optaflexx 45 (Elanco Animal Health, IN, USA) supplemented at 300 mg·animal^−1^·d^−1^ for the final 35 d of the finishing period; LUB: Experior 50 (Elanco Animal Health, IN, USA) supplemented at 36 mg·animal^−1^·d^−1^ for the final 35 d of the finishing period.

3 Initial body weight: the body weight at the beginning of treatment supplementation.

4 SEM = Standard error of the mean.

a,b Means within a row with different superscripts differ, *P* < 0.05.

**Table 5:** Impact of β-ligand supplementation for the last 35 days on feed on reticulorumen pH, temperature, and rumination in finishing beef cattle^1^^,2^. All variables were continuously measured using rumen boluses^3^ that recorded data at 10-minute intervals throughout the entire study period (*n* = 72).

Item	Treatment^4^			SEM^5^	*P*-Value		
	Control	RAC	LUB		Trt^4^	Day	Trt^4^ × Day
***n***	24	24	24				
**Reticulorumen pH **	6.10	6.08	6.14	0.005	0.802	< 0.01	0.170
**Reticulorumen temperature, °C**	39.89	39.86	39.89	0.005	0.279	< 0.01	0.557
**Rumination, min/24 h **	260.39	250.05	250.44	1.443	0.203	< 0.01	0.993

1 Baseline measurements for all variables were collected on d −3, −2, and −1 prior to study initiation, averaged to generate a single baseline value per pen, and included as a covariate in the respective statistical model. Pen was the experimental unit for statistical analysis.

2 Day 34 was removed from the summary and analysis of all bolus related variables; study cattle were transported on this day as part of another experiment and were therefore off feed in the morning and afternoon.

3 SmaXtec pH boluses and Base Station SX.2 (SmaXtec GmbH, Graz, Austria).

4 Treatment: Control: No added β-ligand; RAC: Optaflexx 45 (Elanco Animal Health, IN, USA) supplemented at 300 mg·animal^−1^·d^−1^ for the final 35 d of the finishing period; LUB: Experior 50 (Elanco Animal Health, IN, USA) supplemented at 36 mg·animal^−1^·d^−1^ for the final 35 d of the finishing period.

5 SEM = Standard error of the mean.

### Mobility and behavior

No steers had a mobility score of 3 or greater. There was no treatment by week interaction (*P *= 0.98), treatment (*P *= 0.60), or week (*P *= 0.08) effect on cattle mobility scores (Table 6). The majority of cattle (≥ 89%) had normal mobility across all weeks, and only a small portion (3–11%) of cattle had a mobility score of 2.

**Table 6:** Logistic regression of mobility scores^1^ in finishing beef cattle following β-ligand supplementation over the last 35 days of feeding, across treatment and time point.

Timepoint^2^	Treatment^3^			SEM^4^	*P*-Value		
	Control	RAC	LUB		Trt^3^	Week	Trt^3^ × Week
**Mobility Score 1, %**							
** Baseline **	93.33	91.67	89.09	1.35	0.60	0.08	0.98
** Week 1 **	96.36	95.00	95.00	0.84			
** Week 3 **	94.17	94.91	95.00	1.09			
** Week 5 **	95.83	95.32	94.17	1.16			
**Mobility Score 2, %**							
** Baseline **	6.67	8.33	10.91	1.35	0.60	0.08	0.98
** Week 1 **	3.64	5.00	5.00	0.84			
** Week 3**	5.83	5.09	5.00	1.09			
** Week 5**	4.17	4.68	5.83	1.16			

1 Mobility scores were assessed using the North American Meat Institute (NAMI) scoring system. The definitions for each score are as follows: 1 = Normal, walks easily, no apparent lameness, no change in gait; 2 = Exhibits minor stiffness, shortness of stride, slight limp, keeps up with normal cattle; 3 = Exhibits obvious stiffness, difficulty taking steps, obvious limp, obvious discomfort, lags behind normal cattle; 4 = Extremely reluctant to move even when encouraged, statue-like. No cattle scored a 3 or greater.

2 Timepoint: baseline measurements were collected on d −1 before treatment initiation.

3 Treatment: Control: No added β-ligand; RAC: Optaflexx 45 (Elanco Animal Health, IN, USA) supplemented at 300 mg·animal^−1^·d^−1^ for the final 35 d of the finishing period; LUB: Experior 50 (Elanco Animal Health, IN, USA) supplemented at 36 mg·animal^−1^·d^−1^ for the final 35 d of the finishing period.

4 SEM = Standard error of the mean.

**Figure 1:**
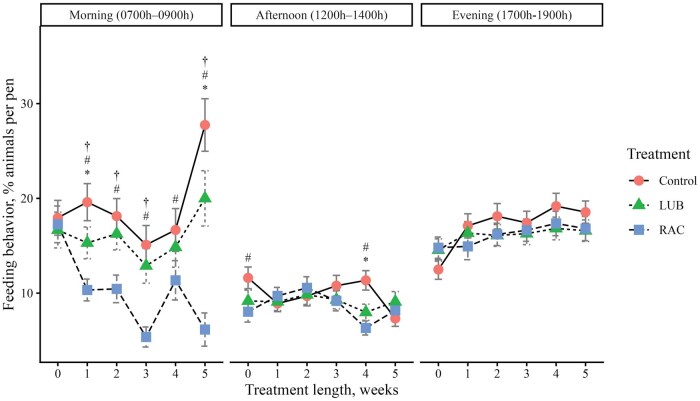
The percentage of animals performing feeding behavior across each week of the study (0 = baseline) by the 3 time periods in the day (Morning, 0700 h to 0900 h; Afternoon, 1200 h to 1400 h; Evening, 1700 h to 1900 h). Feeding behavior was recorded when the steer was facing the feed bunk and was within one body length of the feed bunk. Treatments were (1) Control: no β-ligand, (2) RAC: Optaflexx 45 (Elanco Animal Health, IN, USA) supplemented at 300 mg·animal^−1^·d^−1^, and (3) LUB: Experior 50 (Elanco Animal Health, IN, USA) supplemented at 36 mg ·animal^−1^·d^−1^. Unique symbols (*, #, †) represent significant differences (*P* < 0.05) between treatment by week and time period, where * is the comparison between control and LUB, # is the comparison between control and RAC, and † is the comparison between LUB and RAC.

**Figure 2:**
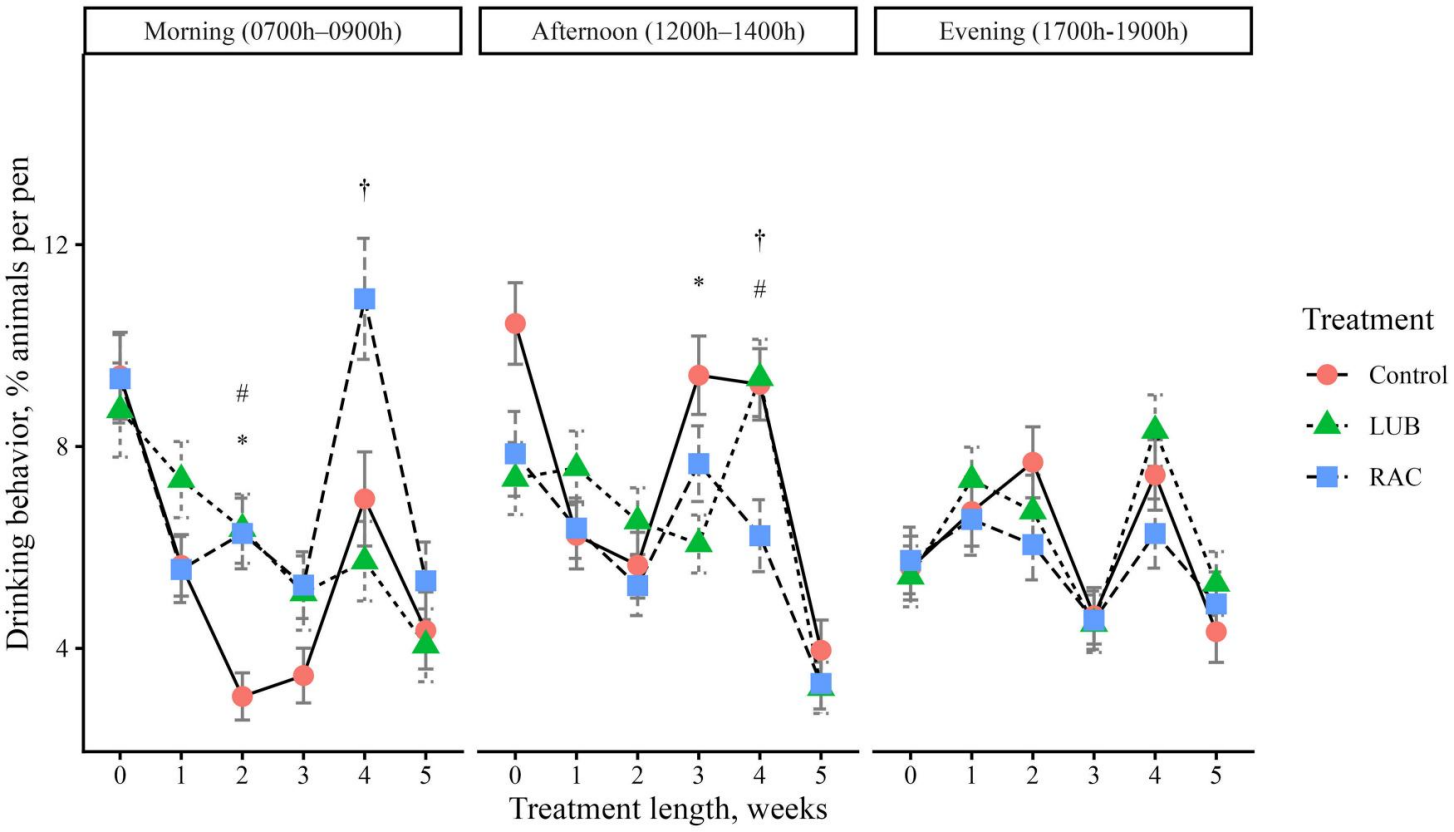
The percentage of animals performing drinking behavior across each week of the study (0 = baseline) by the 3 time periods in the day (Morning, 0700 h to 0900 h; Afternoon, 1200 h to 1400 h; Evening, 1700 h to 1900 h). Drinking behavior was defined as the steer facing the water trough and within one body length of the water trough. Treatments were (1) Control: no β-ligand, (2) RAC: Optaflexx 45 (Elanco Animal Health, IN, USA) supplemented at 300 mg animal^−1^·d^−1^, and (3) LUB: Experior 50 (Elanco Animal Health, IN, USA) supplemented at 36 mg per animal^−1^·d^−1^. Unique symbols (*, #, †) represent significant differences (*P* < 0.05) between treatment by week and time period, where * is the comparison between control and LUB, # is the comparison between control and RAC, and † is the comparison between LUB and RAC.

**Figure 3:**
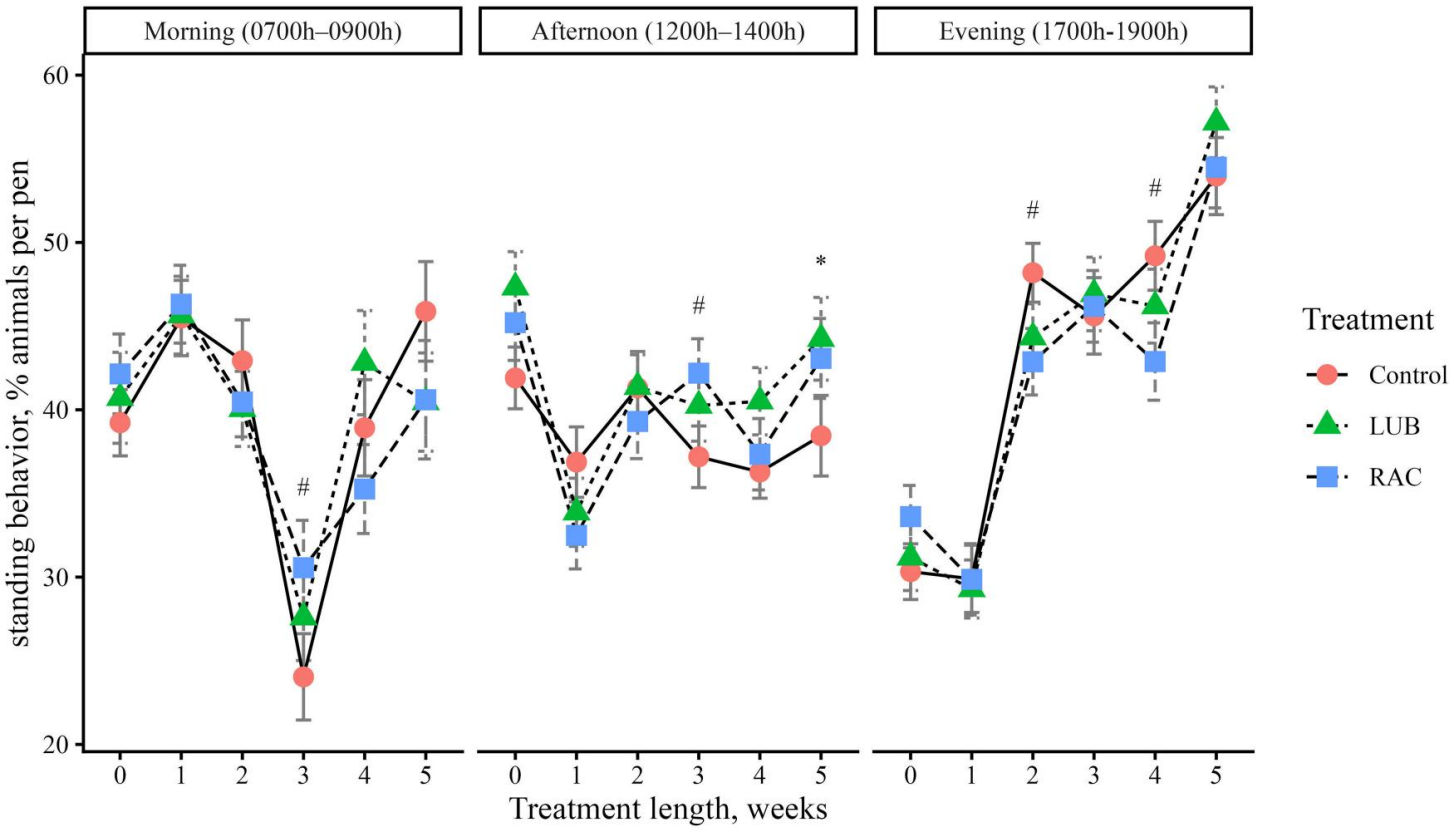
The percentage of animals performing standing behavior across each week of the study (0 = baseline) by the 3 time periods in the day (Morning, 0700 h to 0900 h; Afternoon, 1200 h to 1400 h; Evening, 1700 h to 1900 h). Standing behavior was defined as steer in an upright position supported by all four legs. Treatments were (1) Control: no β-ligand, (2) RAC: Optaflexx 45 (Elanco Animal Health, IN, USA) supplemented at 300 mg·animal^−1^·d^−1^, and (3) LUB: Experior 50 (Elanco Animal Health, IN, USA) supplemented at 36 mg ·animal^−1^·d^−1^. Unique symbols (*, #, †) represent significant differences (*P* < 0.05) between treatments by week and time period, where * is the comparison between control and LUB, # is the comparison between control and RAC, and † is the comparison between LUB and RAC.

**Figure 4:**
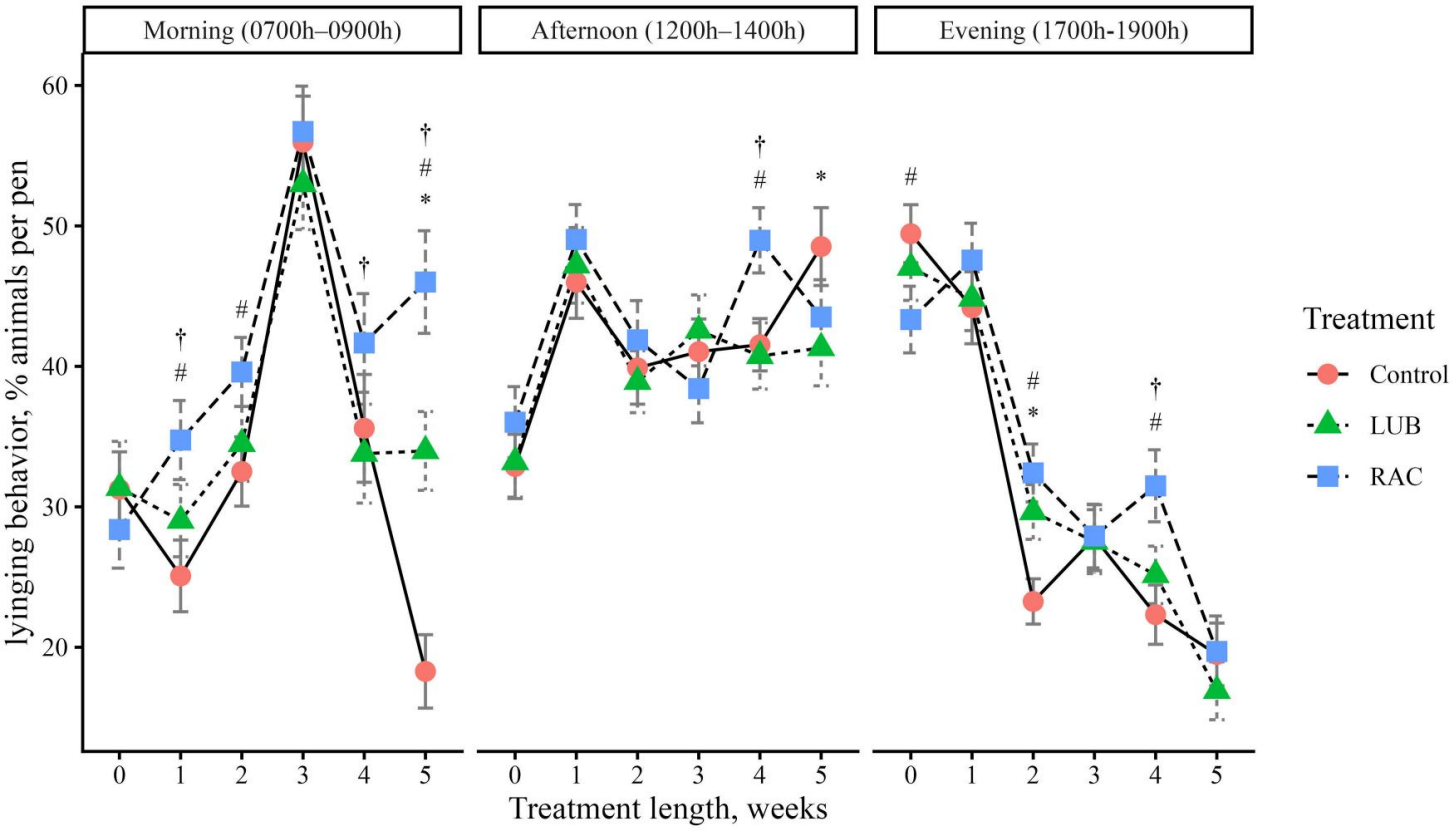
The percentage of animals performing lying behavior across each week of the study (0 = baseline) by the 3 time periods in the day (Morning, 0700 h to 0900 h; Afternoon, 1200 h to 1400 h; Evening, 1700 h to 1900 h). Lying behavior was defined as steer in a recumbent position not supported by its legs. Treatments were (1) Control: no β-ligand, (2) RAC: Optaflexx 45 (Elanco Animal Health, IN, USA) supplemented at 300 mg ·animal^−1^·d^−1^, and (3) LUB: Experior 50 (Elanco Animal Health, IN, USA) supplemented at 36 mg ·animal^−1^·d^−1^. Unique symbols (*, #, †) represent significant differences (*P* < 0.05) between treatments by week and time period, where * is the comparison between control and LUB, # is the comparison between control and RAC, and † is the comparison between LUB and RAC.

**Figure 5:**
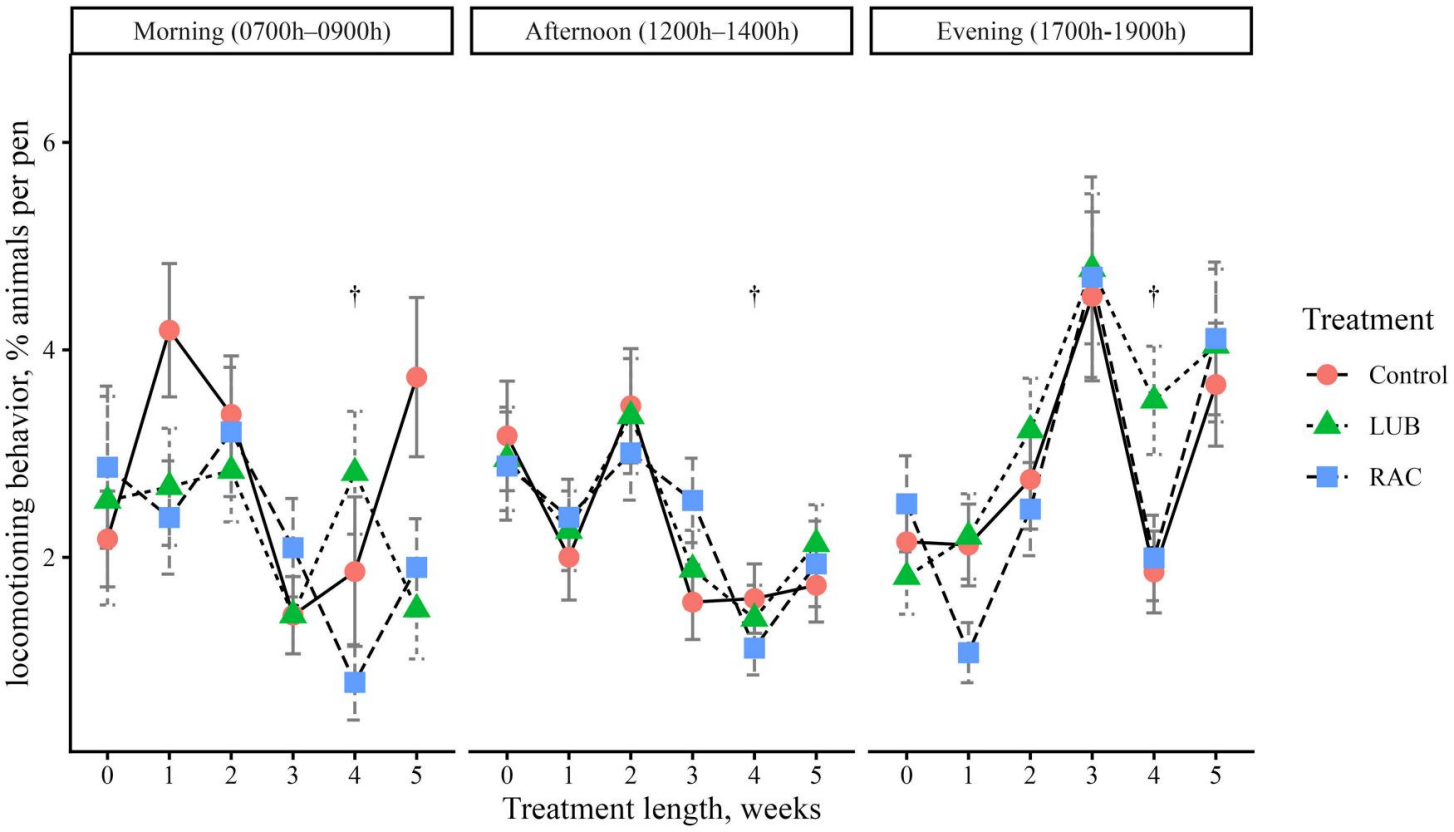
The percentage of animals performing locomotion behavior across each week of the study (0 = baseline) by the 3 time periods in the day (Morning, 0700 h to 0900 h; Afternoon, 1200 h to 1400 h; Evening, 1700 h to 1900 h). Locomotion behavior was defined as steer taking at least two consecutive steps to move its body forward or backward. Treatments were (1) Control: no β-ligand, (2) RAC: Optaflexx 45 (Elanco Animal Health, IN, USA) supplemented at 300 mg ·animal^−1^·d^−1^, and (3) LUB: Experior 50 (Elanco Animal Health, IN, USA) supplemented at 36 mg ·animal^−1^·d^−1^. Unique symbol (†) represents significant differences (*P* < 0.05) between LUB and RAC by week.

There was a 3-way interaction (*P *< 0.01) between treatment, week, and time period on feeding, drinking, standing, and lying behaviors (Figures 1–4). A greater proportion (*P *< 0.05) of cattle from the LUB group exhibited feeding behavior in the morning in weeks 1, 2, 3, and 5 compared to the RAC steers (Figure 1). More cattle (*P *< 0.01) from the control group exhibited feeding behavior compared to the RAC group in the morning in all weeks, and in the afternoon in weeks 0 and 4. Fewer (*P *< 0.01) cattle in the LUB group were eating compared to the control group in the morning of week 1 and in the afternoon of week 4. A lesser (*P *< 0.01) percentage of cattle from the control group were drinking in the morning of week 2 compared to the LUB and RAC groups, and the RAC group had a greater (*P *= 0.003) proportion of cattle exhibiting drinking behavior in the morning of week 4 compared to the LUB group. In the afternoon, the control group had a greater proportion (*P *< 0.01) of cattle that were drinking compared to the LUB group in week 3, and the control and LUB groups had more cattle (*P *< 0.05) that were drinking compared to the RAC group in week 4. A greater proportion (*P *< 0.05) of cattle from the RAC group were standing in the morning and afternoon of week 3, but more cattle (*P *< 0.05) from the control group were standing in the evening of weeks 2 and 4 of the trial. A greater proportion of cattle (*P *= 0.03) from the LUB group were standing in the afternoon of week 5 compared to the control group. A greater (*P *< 0.01) proportion of LUB cattle were lying in the morning of week 5 and evening of week 2, while more cattle (*P *= 0.006) from the control group were lying in the afternoon of week 5. A lesser (*P *< 0.05) proportion of cattle from the control group were lying in the morning of weeks 1, 2, and 5, the afternoon of week 4, and the evening of weeks 2 and 4 compared to the LUB group. A greater proportion (*P *< 0.05) of cattle from the RAC group were lying in the morning of week 1, 4, and 5 and the afternoon and evening of week 4 compared to the LUB group. There was no 3-way interaction (*P *= 0.06) detected in locomotion behavior, but a 2-way interaction (*P *< 0.01) between treatment and week was present (Figure 5). A greater proportion (*P *< 0.01) of cattle from the LUB group were moving compared to the RAC group in week 4 of the treatment period.

### Carcass traits

Table 7 shows the influence of β-ligand supplementation for the last 35 d on feed on carcass characteristics of beef cattle finished in the present study. Hot carcass weight and longissimus muscle area differed by treatments (*P *< 0.01). Steers that received RAC and LUB had greater (*P *< 0.05) HCW and longissimus muscle area than cattle in the control treatment, but there were no differences (*P *> 0.05) in these parameters between cattle provided RAC and LUB. There were no differences (*P *> 0.05) in dressing percentage, quality grade, marbling score, subcutaneous fat depth, USDA yield grade, and color between control, RAC, and LUB cattle. All carcasses fell within the normal color category.

**Table 7:** Impact of β-ligand supplementation for the last 35 days on feed on carcass characteristics and muscle color in beef cattle finished in the summer.^1^

Item	Treatment^2^			SEM^3^	*P*-Value
	Control	RAC	LUB		
**Pens/treatment **	12	12	12	—	—
**Animals/treatment **	120	120	120	—	—
**Hot carcass weight, kg **	449.5^b^	455.2^a^	457.4^a^	1.84	<0.01
**Dressing percentage^4^**	63.0	63.4	63.6	0.23	0.59
**Quality grade^5^, %**					
** Choice**	82.2	86.2	81.5	0.02	0.57
** Prime**	17.8	13.8	18.5	0.02	0.57
**Marbling score^6^**	616.6	618.6	605.6	10.40	0.73
**Subcutaneous fat depth, cm **	1.83	1.76	1.80	0.08	0.66
**Longissimus muscle area, cm^2^**	95.89^b^	99.10^a^	100.54^a^	0.85	<0.01
**USDA yield grade **	3.35	3.14	3.08	0.10	0.06
**Color^7^**	76.6	77.0	76.5	0.93	0.11

1 Initial body weight was used as a covariate for statistical analysis of all response variables.

2 Treatment: Control: No added β-ligand; RAC: Optaflexx 45 (Elanco Animal Health, IN, USA) supplemented at 300 mg·animal^−1^·d^−1^ for the final 35 d of the finishing period; LUB: Experior 50 (Elanco Animal Health, IN, USA) supplemented at 36 mg·animal^−1^·d^−1^ for the final 35 d of the finishing period.

3 SEM = Standard error of the mean.

4 Dressing percentage was calculated by applying a 4% pencil shrink to the final live body weight.

5 Values represent the predicted probability (in percentage) that carcasses from each treatment were assigned to the quality grade.

6 Marbling score: 300 = Slight, 400 = Small, 500 = Modest, 600 = Moderate, 700 = Slightly Abundant (USDA 2006).

7 Color score of < 55 indicated a ‘Dark Cutter,’ scores between 55 and 59 represented ‘Off-Colored’ meat, and scores ≥ 60 were classified as ‘Normal’ meat color.

a,b Means within a row with different superscripts differ, *P* < 0.05.

## Discussion

Heat stress has been a major issue faced by the feedlot cattle industry that negatively impacts animal production, welfare, and return on investment for producers, estimated to cost the industry millions of dollars annually (St-Pierre et al. 2003; O’Brien et al. 2010; Kubik et al. 2018). The potential link between β-AA and severe heat stress-related symptoms has raised some concerns among industry stakeholders about β-ligand supplementation in the summer. In addition, a newer β-ligand molecule like LUB, with its unique mode of action, has not been well studied on its impact on cattle welfare during times of heat stress. The current study was conducted during the summer months in Colorado, so that cattle would be experiencing a hotter environment with the potential to experience heat stress, which would allow for the evaluation of the impact of β-ligand supplementation during the summer months on feedlot cattle. Indices such as THI and HLI, can be used to set thresholds beyond which the risk that cattle will experience thermal stress increases (Herbut et al. 2018). In the current study, although cattle experienced an average ambient temperature within their thermoneutral zone, they did experience significant extreme temperatures, particularly during the midday hours. Although on average across time periods, the THI was within the comfort zone during all times of day, approximately 15% of THI was above the thermoneutral zone (THI > 75). The HLI, as proposed by Gaughan et al. (2008), is another index that considers BGT, RH, and windspeed, which is not included in the THI calculation. Silva et al. (2007) evaluated several heat load indices and found that HLI was significantly correlated with both rectal temperature and respiration rate. Gaughan et al. (2008) indicated that when HLI > 86, black-hided unshaded cattle, as was the case in the current study, would start to accumulate heat. Again, although average HLIs were well below this value, HLI exceeded the thermoneutral zone (HLI > 70) for 1.4% of the observation period, and exceeded 86 (very hot) for 0.4% of the time, suggesting that cattle were periodically accumulating heat. Additionally, depending on the duration of high heat load conditions and the ability of cattle to cool and dissipate heat during the evening hours, cattle may experience heat stress at lower HLIs. In short, during the current study, cattle were at risk of experiencing heat stress intermittently based on several of the measured environmental parameters.

The performance results of the current study further support the findings from previous studies (Avendaño-Reyes et al. 2006; Abney et al. 2007; Gruber et al. 2007; Kube et al. 2021; Teeter et al. 2021; Edmonds et al. 2024), that β-ligands enhance feed efficiency and lean muscle gain. In general, cattle that received RAC and LUB had improved growth performance and carcass characteristics compared to the cattle that did not receive β-ligands in the current study. These findings indicate that both β-ligands were effective in enhancing production outcomes when fed in the summer months in Colorado, with no major differences observed between RAC and LUB in the current study. In contrast, a previous study reported a greater final BW, ADG, G:F, and HCW in LUB-supplemented steers compared to steers receiving RAC (McAtee et al. 2024). Another study conducted by Shreck et al. (2024) in Beef × dairy crossbred steers also observed greater ADG, G:F, HCW, DP in steers fed LUB compared to RAC supplemented steers. Similar to the current study, Wendler et al. (2025) did not observe differences in feedlot performance between RAC and LUB cattle that had similar days on feed, but they observed greater HCW and dressing percentage in steers that received LUB compared to steers receiving RAC. The discrepancy between the growth response and HCW differences between RAC and LUB supplemented steers reported by previous studies and the current experiment may be due to differences in the β-ligands’ feeding duration (McAtee et al. 2024; Wendler et al. 2025). In the current study, RAC and LUB were both fed for 35 d before slaughter, but McAtee et al. (2024) supplemented RAC for 28 d and supplemented LUB for 56 d, while Wendler et al. (2025) supplemented RAC for 35 d and LUB for 56 d before slaughter. The improvement in growth performance and carcass characteristics in LUB cattle in those experiments could be due to the longer LUB feeding duration compared to RAC cattle. In the current experiment, the marbling score was not impacted by beta ligand feeding for 35 d. Edmonds et al. (2024) reported that dose and duration of Lubabegron can impact marbling scores in feedlot cattle. Feeding Lubabegron at 1.5, 3.5, and 5.5 mg/kg DM for 56 days or longer reduced marbling score regardless of dose. In the current experiment, Lubabegron (3.1 mg Lubabegron/kg DM) was fed for 35 days, which may not have been long enough to decrease marbling scores. Whereas feeding ractopamine hydrochloride at 100–400 mg/kg DM for 14-, 28, 35, or 42 days had no impact on marbling scores in steers and heifers (Bittner et al. 2016; Edenburn et al. 2016; Bittner et al. 2017), which is consistent with our findings.

In addition to growth and carcass outcomes, it was also important to determine if β-ligand supplementation impacted rumen fermentation, which could potentially impact heat stress response due to the change in metabolic heat generation. The current study showed that the supplementation of RAC and LUB did not alter rumen fermentation characteristics, as reticulum pH, reticulum temperature, and rumination did not differ between the β-ligand supplemented steers and control steers. The mode of action of β-ligands is known to alter the biological processes within particular cells (such as skeletal muscle cells and fat cells) to promote more lean muscle production (Johnson et al. 2014; Pfau et al. 2023), but the similarity in rumination between cattle fed β-ligands and negative control groups has not been evaluated previously. In addition, control and β-ligand supplemented steers had similar DMI in the current experiment, which could also explain the similarity in rumination parameters measured in this study.

To evaluate the impact of β-ligand supplementation in the summer on animal welfare, cattle in-pen mobility and in-pen behavior were also measured in the current study. Cattle mobility has been an important indicator of animal welfare and is now one of the measurements included in the National Cattlemen’s Beef Association’s National Beef Quality Audit (Eastwood et al. 2017). In the current study, cattle mobility scores did not differ between steers with or without β-ligand supplementation despite the differences in final BW. The majority of cattle (≥ 89%) in the current study scored a mobility score of 1 (normal), and no steer had a score of 3 or higher. Like the current study, mobility scores of cattle in past studies did not differ between LUB vs. negative control (Kube et al. 2021) and LUB vs. RAC (McAtee et al. 2024). In addition, Edmonds et al. (2024) measured feedlot cattle mobility at loading prior to transport to the slaughter plant and reported that ≥ 95% of the cattle, independent of LUB dose, exhibited normal mobility. Similarly, Wendler et al. (2025) reported that ≥ 93% of cattle had normal mobility, independent of β-agonist type and LUB duration, when mobility was measured at the slaughter plant. As noted in previously published studies (González et al. 2012; Edwards-Callaway et al. 2017; Lee et al. 2018; Mijares et al. 2021), cattle mobility can be impacted by multiple factors, including but not limited to animal characteristics, location of scoring (i.e., feedyard vs. plant), sex, weather, body weight, transport conditions, and feeding management.

This study evaluated in-pen behavior during the supplementation period, and interactions between treatment, week, and time of day were observed. During morning observations, a greater proportion of cattle in the control group were engaged in feeding behavior as defined in this study (which also included animals in close proximity to the feed bunk). In comparison, the RAC steers had the least proportion of steers feeding versus LUB and control steers. Cattle behavior varies across times of the day (Kilgour 2012); thus, differences across different sampling periods would be expected. Trotta et al. (2019) reported that cattle fed RAC exhibited decreased time eating per visit, per meal, and per day as compared to cattle fed no β-ligand. Interestingly, the authors measured feeding behaviors using an electronic feeding system and found that the RAC-fed animals had a greater DMI consumption per minute as compared with the control animals (Trotta et al. 2019). In contrast, Abney et al. (2007) reported that RAC-fed cattle took longer to consume their ration as compared to non-supplemented cattle. There are many different methods employed to assess behavior, and sometimes comparisons between studies are challenging. As noted, feeding behavior in LUB-fed cattle has not been evaluated previously, and thus there is an opportunity to further explore feeding behavior in future work.

An opposite trend was seen with lying behavior. The RAC group was lying compared to the control group in the morning. Stackhouse-Lawson et al. (2015) reported that cattle fed zilpaterol hydrochloride exhibited similar total lying time as compared to non-supplemented cattle. However, zilpaterol-fed cattle exhibited increased lateral lying behavior during the feeding period of 21 d prior to slaughter. The current study did not differentiate between lying types (i.e., lateral versus sternal lying), but this could be included in future work with a different sampling approach. Changes in lying behavior across treatments were also seen during different times of day, but a treatment-specific pattern was not observed across all time periods and weeks. Although the direct impact of ground temperature on lying behavior was not quantified in the current study, it should be noted that ground temperatures during the midday behavior observation period averaged almost 40 °C, perhaps influencing the percentage of cattle lying during this time period. During hot conditions, cattle stand more as a mechanism to dissipate more heat (Galán et al. 2018). In the current study, the percentage of cattle standing changed across treatment, time periods, and treatment length, but one clear trend related to the proportion of cattle standing and time of day was not observed. A study exploring behavioral changes of feedlot cattle housed in a climate-controlled facility reported that cattle spent a lesser proportion of time lying and a greater proportion of time standing during the hot period of the study (Idris et al. 2024); the THI during the study ranged from 66.4 and 84.8, similar to what was observed in the current study. In the current study, behavior was only observed for 3 time periods on one day per week; future work could expand upon the amount of time behavior is observed specifically during hotter times of day to understand how cattle adapt behavior to combat increasing ambient and ground temperatures.

Although some differences in drinking behavior were observed between treatment groups, no specific trend was detected. Previous literature reported an increase in water intake by feedlot cattle when the temperature increased, and reported greater water consumption in the summer compared to the winter (Hoffman and Self 1972; Arias and Mader 2011). It is important to note that the current study evaluated drinking behavior by instantaneous sampling methods of a 10-minute scan interval and recording the number of cattle that were drinking and those that had their heads over the waterer. The interpretation of drinking frequencies related to total heat load and treatments may be limited, as total water intake was not measured in the current study.

## Conclusion

The utilization of β-ligands to promote lean muscle gain and improved feed efficiency in the cattle industry has been well established. The introduction of a molecule like LUB to reduce ammonia gas, along with published studies indicating its ability to improve feed efficiency and carcass weight, presents opportunities to advance the sustainability of beef production. This study explored the potential of β-ligand supplementation in improving the overall production of cattle finished during the summer months. In the current study, cattle supplemented with RAC and LUB exhibited improved feedlot performance and carcass characteristics compared with cattle that did not receive a β-ligand. Supplementation of β-ligands did not impact rumination, cattle mobility, or cattle in-pen behavior in the current study. While these findings support the efficacy of β-ligands in enhancing cattle productivity, the potential impact of extreme heat load on their mode of action was not seen in this study, where mild heat stress was experienced. Continued research is needed to understand how severe and sustained ambient temperatures, that cause heat stress in feedlot cattle, interact with β-ligand supplementation.

## Abbreviations

AIC: Akaike’s information criterion
AT: ambient temperature
AV: anterior ventral
ADG: average daily gain
β: beta
β-AA: beta-adrenergic agonists
β-AR: beta-adrenergic receptor
BGT: black globe temperature
BW: body weight
cAMP: cyclic adenosine monophosphate
DMI: dry matter intake
FDA: U.S. Food and Drug Administration
G:F: gain:feed ratio
HLI: heat load index
HCW: hot carcass weight
LUB: lubabegron
NASEM: National Academies of Sciences, Engineering, and Medicine
NAMI: North American Meat Institute
PKA: protein kinase A
RAC: Ractopamine Hydrochloride
RH: relative humidity
TMR: total mixed ration
THI: temperature–humidity index
Trt: treatment
WS: wind speed
